# Print, Eat, Heal: Unravelling the Potential of Bioactives in 3D Food Technology

**DOI:** 10.3390/foods15020260

**Published:** 2026-01-11

**Authors:** Monize Bürck, Monica Masako Nakamoto, Sergiana dos Passos Ramos, Marcelo Assis, Anna Rafaela Cavalcante Braga

**Affiliations:** 1 Department of Biosciences, Universidade Federal de São Paulo (UNIFESP), Santos 11015-020, Brazil; monize.burck@unifesp.br (M.B.); monica.nakamoto@unifesp.br (M.M.N.); sergiana.passos@unifesp.br (S.d.P.R.); marcelostassis@gmail.com (M.A.); 2 Postgraduate Program in Nutrition, Universidade Federal de São Paulo (UNIFESP), São Paulo 04023-062, Brazil; 3 Nutrition and Food Service Research Center, Universidade Federal de São Paulo (UNIFESP), Santos 11015-020, Brazil; 4 Department of Chemical Engineering, Universidade Federal de São Paulo (UNIFESP), Diadema 09920-000, Brazil

**Keywords:** 3D technologies, antioxidant activity, bioaccessibility, innovative ingredients, food industry

## Abstract

3D-printed food (3DPF) is on the rise, enabling the development of new food products. Current applications in this domain led to the replication of meat analogs, protein-enriched products, and dietary solutions tailored to address nuanced health necessities. Central to the functional versatility of 3DPF is its capacity for post-printing textural manipulation, which facilitates diverse food applications. Integrating bioactive compounds sourced from biodiversity, vegetables, algae, and agricultural residues is not merely an exercise in culinary refinement but an outstanding contribution to the circular economy. Strategic incorporation of these bioactive compounds into foodinks enhances the antioxidant potential of consumables and contributes to physiological benefits for human health, as evidenced by extant literature, which underscores their antioxidative and anti-inflammatory properties. Nevertheless, critical gaps emerge upon a meticulous examination of the recent literature, notably regarding the viability of bioactive compounds within foodink matrices for 3DPF and their bioaccessibility after simulated digestion. Thus, the objective of this review is to evaluate the current state of the art in 3DPF, with a focus on biodiversity as a source of innovative ingredients and matrices and on the bioaccessibility of associated bioactive compounds, while outlining future research directions in this field.

## 1. Introduction

The 3D printing industry uses a technique for manufacturing three-dimensional objects through Computer-Aided Design (CAD), primarily in the automotive, pharmaceutical, medical, and food industries [1]. In the food sector, target structures are built layer by layer, superimposing consecutive layers of edible materials until the desired 3D-printed food (3DPF) is obtained [2]. Although it is a promising technology, it is in an early phase, with an estimated annual economic impact of US$ 550 billion on the food industry by 2025 [3]. Currently, the concept of 3DPF offers greater creativity, customization, and sustainability, which can revolutionize food innovation [2].

It is believed that including food production in the universe of 3D printing will set new limits when it comes to, in general, the creation of personalized foods [4], their format, cost involved, flavor, and texture, with new shapes without the need to use molds, resulting in reduced production costs and low-waste production [5]. In particular, the development of bioinks for 3DPF has attracted interest from researchers and industry, as their formulations offer numerous opportunities for food customization. Bioinks are substances, usually in a creamy or paste consistency, that contain natural or synthetic biomaterials, either independently or in a hybrid combination [6,7]. In the context of 3DPF, bioinks formulated with edible ingredients will be referred to in this work as foodinks. This distinction separates the terms “3D food printing” and “conventional 3D printing,” with the former using edible formulations that may or may not include bioactive compounds. These biomolecules can exhibit antioxidant activity by scavenging free radicals [8,9,10] and thus protect health against oncogenesis [11,12], inflammatory diseases, hypertension [13], obesity, and hepatic lipid composition when regularly consumed [14]. Therefore, building nutritional composition linked to a relatively simple supply chain expands the source of materials available for such use [15]; likewise, the bioactive compounds from the biodiversity [6,7].

Although there is a growing interest in 3DPF as a technology for the food industry, the existing literature addresses the characterization of the foodinks or the final printed products; however, there is a lack of studies exploring the synergy between bioactive compounds derived from biodiversity and their bioaccessibility, and the other components of the bioinks matrices. Their integration with delivery systems designed to enhance bioaccessibility [16,17] remains insufficiently investigated. Thus, this review aims to evaluate 3DPF’s state of the art, focusing on the potential of biodiversity as a source of innovative ingredients and matrices, and the bioaccessibility of the bioactive compounds. Additionally, it aims to outline the next steps in this field.

## 2. Materials and Methods

A bibliographic search within article title, abstract, and keywords was conducted in the Scopus® database (Elsevier, Amsterdam, The Netherlands) using the syntax “(“3D printing” OR “3D food” OR “3D food printing” OR “printed food”) AND (“bioactive compounds” OR “biomolecules”)”. Only original peer-reviewed papers published from 2021 to 2025 in English were included in the present study, and the review articles were excluded. The resulting 210 original papers were downloaded in .csv format to generate the bib-liometric map using VOSviewer (version 1.6.19, Leiden, The Netherlands) [18]. Then, the syntax “(“3D” AND “food” AND “bioactive compounds”)” was conducted and submitted to the same inclusion/exclusion criteria, resulting in 57 original papers. All selected articles were registered and underwent abstract review. Then, 14 manuscripts were carefully read, and the information was summarized in Section 5. Relevant studies cited by those registered articles were also read, and the main ideas were discussed.

## 3. Bioinks and Foodinks

Bioinks are defined as combinations of living cells, biomaterials, and bioactive substances, and they are among the main components of 3D printing. To promote high-quality 3D printing, attention must be paid to bioinks factors, including printability, mechanical properties (post-deposition strength), and functionality (benefits conferred by their composition to humans and their lack of toxicity to cells) [19]. For the 3D printing process, the formulation of bioinks is essential as it interferes not only with the rheological properties of the extruded material but also assists in mechanical resistance to ensure shape retention after deposition on the platform. Thus, knowledge of the main constituents of bioinks and how their properties influence the printing technique is fundamental to ensuring the quality of the printed product [20].

The fluidity of bioinks can be achieved through plasticization or fusion, and these processes must be evaluated considering the characteristics of the bioinks components, i.e., temperature stability and hydrogel crosslinking [21]. The main components used in the composition of bioinks for 3DPF—specifically foodinks, which are bioinks intended for food applications—are carbohydrates, proteins, lipids, and specific polymers and their fractions, which significantly alter the rheology of the mixture, the melting temperature, and the plasticizing power. When used in appropriate proportions, carbohydrates (monosaccharides, disaccharides, oligosaccharides, and polysaccharides) assist in the extrusion process and help maintain the shape of the formed food structure. An important parameter analyzed when using carbohydrates as foodinks is the glass transition temperature (Tg), which is associated with significant changes in carbohydrate structure, leading to fundamental rheological changes during processing. High-molecular-weight carbohydrates, such as oligo- and polysaccharides, have higher Tg (usually above 100 °C) than low-molecular-weight carbohydrates, such as mono- and disaccharides (usually below 60 °C) [22].

The presence of proteins (such as pectin, whey protein, egg proteins, edible insects, and bean protein) in the matrices used in foodinks affects the texture and structure of the resulting food product. If proteins are the main constituent of the foodink, then pH and isoelectric point are the foremost parameters to be controlled. Due to the positive and negative charges associated with the amino acids that form the protein, adjusting the isoelectric point allows aggregation of the mixture, an essential resource for gelatinization and hydrogel formation, resulting in varied textures for the desired product [3,20]. It is possible to obtain new textures by alternating deposition of layers of protein-based inks with polysaccharide-based inks, such as gelatin and alginate. Additionally, proteins are composed of long chains of amino acids. Due to their long and complex chains, some proteins are not printable; therefore, they must be denatured initially by thermal or mechanical stress or by solid acids or bases to obtain a broader range of three-dimensional structures and improve the printing capacity of these macromolecules [23,24].

Lipids are formed by the esterification of three long-chain fatty acids joined by a glycerol molecule to form triglycerides. The presence of lipids in the composition of foodinks affects the functional properties of the resulting food material, including the melting range and crystalline structure. The structure of fatty acids in lipid formation interferes with their fusion, as long-chain fatty acids generally have high melting points. Additionally, unsaturation in carbon chains affects heat treatment: double bonds result in structures with lower melting points. However, saturated chains result in better product storage stability, as they have melting points of around 30 °C to 40 °C. Thus, the composition of triglycerides can help regulate the melting point of the deposited layers and determine the material’s properties before and after processing [25].

On the other hand, hydrocolloids are hydrophilic polymers that require the addition of water or gelling agents to improve printing capacity. Various carbohydrates (such as methylcellulose, xanthan gum, starch in general, and agar) and proteins (such as gelatin, chitosan, and whey protein), which do not naturally have printing capacity, can be used for hydrocolloid production, allowing understanding of interactions and influence on molecular weight on rheological properties, simulating the printing capacity of cosmetic substances [26]. Hydrogels can be developed as foodinks for 3D printing to improve printing conditions. These compounds have remarkable flexibility, viscoelasticity, and high polarity, providing low surface tension. Combining oleogels and hydrogels allows the incorporation of hydrophobic bioactives without impairing the formed materials [27]. Two bigels were formulated to evaluate their applicability for 3D printing: one containing beeswax, agar, and xanthan gum, and the other containing only agar and gelatin. The results demonstrated that both formulations showed good printing capacity, with the first formulation promoting better printability, an excellent surface, and less variation in the dimensions of the obtained products [28]. Lenie et al. [29] developed a hydrogel combining carbohydrates and proteins (alginate and pectin) at different concentrations; however, only adding calcium chloride increased printability with foodink.

Some studies demonstrate that incorporating foods or bioactive compounds into foodink formulations improves the rheological properties of the ink, enhances printability, and maintains the biological properties of the compounds. Most foodinks developed for 3DPF use carbohydrates or proteins as matrices for incorporating bioactive compounds. Starch is widely used due to its low cost and gelling power. The strawberry extract was combined with corn and wheat starch to form foodink for 3DPF; the results demonstrated the printing capacity of the foodink and that the presence of the bioactive compound significantly altered the material’s rheological properties without impairing the biological properties of the extract [6,30]. Starch can also be used for printing along with hydrophobic bioactive compounds. Lutein was added to a starch gel for 3DPF, and it was observed that the gel provided thermal protection and stability for lutein compared to raw lutein [16].

Soy protein was used together with red cabbage extract to produce a 3DPF. The results demonstrated that the extract altered the foodink extract and decreased the hardness and gumminess of the formed material, making it more desirable for consumption [31]. Gardenia fruit extract was added to a microgel solution containing gelatin, β-cyclodextrin, and chitooligosaccharides to form foodink for 3DPF. The protein/carbohydrate formulation conferred stability of the extract against exposure to UV radiation. The presence of the extract in the foodink indicated high printing potential, shape retention, and maintenance of the formed structure.

Foodink containing wheat or corn starch loaded with *Arbutus unedo* fruits would provide, on average, 632.60 ± 6.12 of total phenolic compounds. However, the authors have not assessed the bioaccessibility of these compounds to better understand their release from the foodink matrix [32], regarding rheological parameters, samples containing corn starch required lower force (N) during extrusion, indicating that the other ingredients directly influence printability and 3DPF results.

Given the above, it is notable that the formulation of foodinks alters rheological properties and confers essential characteristics to the resulting material, such as mechanical resistance and shape retention. The choice of biomolecule is determined by the desired properties of the final product, with a focus on sensory functions. The addition of bioactive compounds to the foodink promotes improvements mainly in the ink’s rheology, and evaluating the maintenance of these compounds’ biological activities is fundamental, since the 3D printing process imposes mechanical and thermal stresses on the solutions.

## 4. Overview of 3D Food Printing

Investing time, money, and new technologies in this field means reaching more consumers based on their ages, sexes, occupations, and lifestyles, and adjusting compositions, rigidity, and structures to the client’s needs and preferences. Consumers’ choice to purchase food is guided by the following criteria: taste, cost, experience, convenience, and nutrition. 3DPF can satisfy all these criteria and manufacture personalized/customized food for general or specific consumer groups (children, older people, pregnant women, teenagers, athletes, vegans, vegetarians, etc.) concerning sensory and nutritional properties. Moreover, hundreds of innovative foods in terms of shape, dimensions, consistency, microstructure, color, taste, flavor, etc., could be obtained by printing blends or by multi-printing several food ingredients [27,33,34,35,36,37].

**Figure 1:**
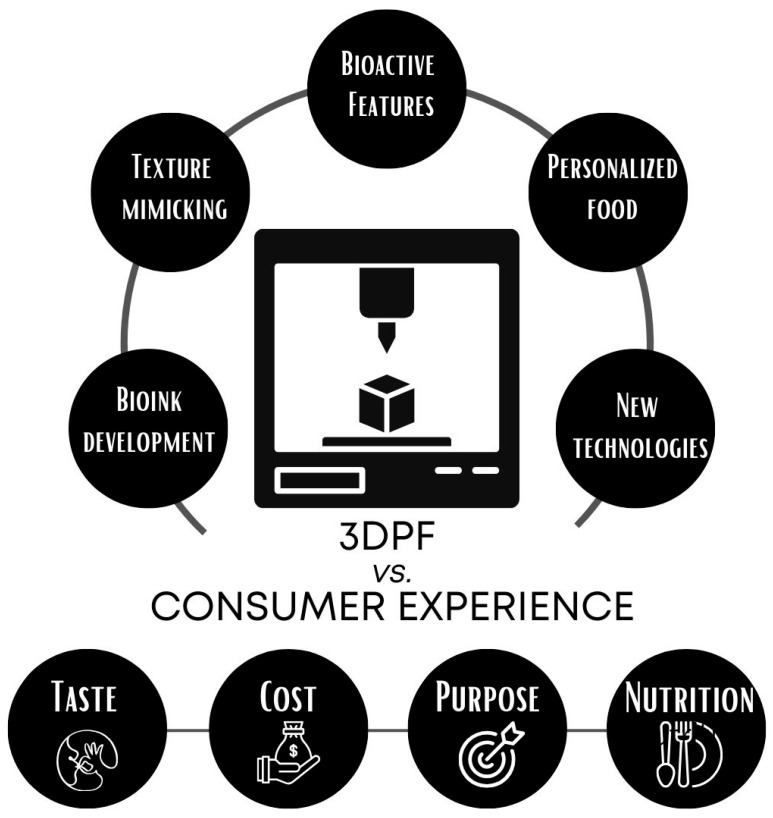
The relationship between the 3DPF and the consumer experience.

Considering the possibilities and pathways for integrating bioactive compounds into 3DPF and the need for physicochemical assessment, it is crucial to consider the potential implications of their consumption for human health, as shown in Figure 1.

**Figure 2:**
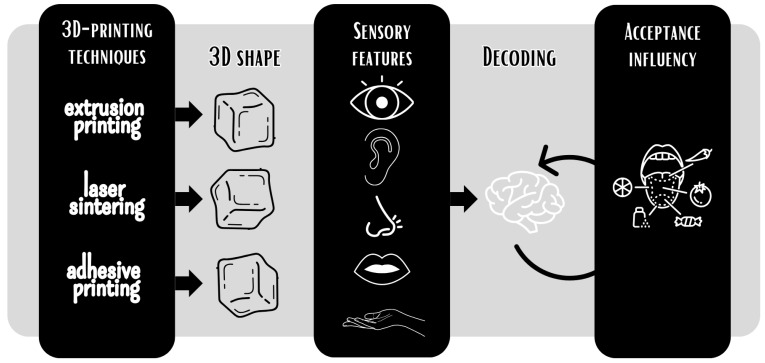
How the printing technique can influence the shape and mechanical properties of 3DPF and change consumer sensory perceptions.

Although three methods to obtain 3DPF can be highlighted (Figure 2), the most used procedure is extrusion, which involves foodinks usually composed of gels or pasty materials smoothly extruded through the printer nozzle, being able to receive bioactive compounds and manage to maintain the desired shape of the product, in addition to the components tailored for the base [38]. Thus, the viscosity and rheological properties of the biomaterial are the primary focus [39]. Specific process parameters to be considered in this method are the nozzle’s height at the deposition time, its diameter, and its movement speed [15]. Another critical parameter is the material’s capacity to be deposited and to maintain its structure, i.e., its printability.

Direct ink writing (DIW) is an extrusion-based (Figure 2) method where the foodink is deposited layer by layer as continuous filaments with sufficient viscoelasticity and printability, e.g., apple pureed in combination with cellulose nanocrystals (CNC) [40] or powdered milk with no additives, only deionized water [41], demonstrating the versatility of the DIW method. The following extrusion-based techniques are selective laser sintering (SLS) and adhesive printing (AP) (Figure 2). These methods allow the design of shapes and the control of the mechanical properties of the final products. In SLS, a layer of powdered ingredients is fused by a laser in a sequential process, enabling control of the microstructure of the 3DPF [42]. In contrast, in AP, the first layer is also a powdered ingredient that is repeatedly sprayed with a binder liquid, such as water, to join the powder and form confectionery or snacks [43,44]. All these methods may shape consumers’ perceptions (Figure 1 and Figure 2).

The post-processing of the 3DPF is equally essential as printability. This factor depends on rheological, physical-chemical, and mechanical properties; post-processing encompasses how the final product will resist practices commonly applied to food, such as oven or vacuum drying, steaming, air frying, and so forth. In this regard, to develop new strategies for 3DPF printing, it is clear that pre- and post-printing factors must be defined to inform the selection of raw materials and the development of foodinks. Still, depending on the application, contamination of 3DPF structures must be avoided at all costs in post-printing care. The same precautions that must be taken at any stage of food processing must be considered after 3D printing. Many printers even have UV lamp systems that can be activated to eliminate microbial forms that may be present in printed products.

### 4.1. Presentation Coupled with Acceptance: Using Less Conventional Foods, Even Those Aesthetically Imperfect

Commonly consumed food has been applied to 3DPF. Chocolates [45,46,47,48], cookies [49,50,51], gummies [52,53,54,55], and cheese [56,57]. 3DPF aims to introduce innovations to recipes and improvements to technological processes, as well as to develop new products in response to specific consumer demands. Notably, at least two recent studies investigated the sensorial perception of chocolate 3DPF in different shapes. They concluded that more complex shapes were more accepted [58] and that the shape influenced the perception of the sweet taste but not of the sourness and bitterness [57].

These findings support the use of alternative ingredients for foodink production, including algae, fungi, insects, seaweed, roots, and imperfect vegetables [59], to reduce food waste [3] and promote the circular economy in food production, coupled with 3DPF. Worldwide, around 2.3 billion tons of food are wasted annually; this loss is one of the leading and most critical obstacles to sustainable development. Additionally, the term “low cost” is only weakly associated with bioactive compounds, once again encouraging the exploration of pre-existing resources to control costs and promote sustainability.

To minimize the impact of this problem, different 3D food printing technologies have been used to utilize wasted food [29,59]. These technologies are applied to produce food, food packaging, medical consumables, personalized/custom-made products, semi-structural load-bearing applications, customization, and high-precision manufacturing (Figure 2). DIW technology is the most widely used to upcycle materials in 3DPF, mainly employing rice husk, fruit peels, okara, spinach stems, kale stalks, sugarcane bagasse, gelatin, and alginate in its formulations [60].

### 4.2. Innovation Leads to Meat Analogs: The Pursuit of Perfect Texture and Presentation Has Begun

Investment in the production of alternative foods is on the rise, driven by society’s concern for healthier options and consumer demand for a sustainable food chain. This results in lifestyle changes, primarily reflected in the significant increase in the vegetarian and vegan populations worldwide. Developing foods such as meat, chicken, and fish has become widespread, using high-biological-value proteins and nutrients derived from algae and insects [2,8,49,61].

Innovatively, optimal formulation of foodink for chicken meat-based noodles containing 6% oat bran and 2% konjac flour achieved the best 3D printability [62]. Fiber from oats and konjac flours improved dough viscosity and viscoelasticity. However, konjac flour at >4% resulted in high water absorption and negatively affected the final quality of the products. As expected, fiber-enriched noodles increased the time required for starch/protein digestion. The results suggest that konjac flour may form networks around protein molecules, leading to their poor digestibility, underscoring the importance of further research on texture agents. Furthermore, mushroom cultivars (saffron milk-cap, *Lactarius deliciosus*; reishi, *Ganoderma lucidum*; oyster, *Pleurotus ostreatus*) in foodinks were evaluated for their rheological properties, color, texture, cooking loss, and amino acid content, and sensory analysis was assessed on the printed samples. All the inks showed shear-thinning and gel-like viscoelastic behavior [63]. The plant-based meat analogs were successfully printed, and the incorporation of mushrooms reduced stiffness, elasticity, and chewiness and increased juiciness, better nutritional value, and the release of umami amino acids, resulting in reasonable overall acceptance compared to the control. Meat analog structures coupled to extrusion methods of 3DPF using textured pea protein fibrils, locust bean gum hydrocolloid, and/or sodium alginate, and microbial single-cell protein were also reported [64].

The overview of 3DPF highlights the technology’s potential to influence meat texture, leading to meat analogs that are more widely accepted, even among individuals who do not strictly adhere to vegetarian or vegan diets. The rheological analysis approach contributes to optimizing the printing process, achieving desired textural attributes in the final 3D-printed meat analogs, and improving the digestibility of the foods. As the search for innovative solutions in this domain continues, a deeper exploration of rheological properties will significantly help in developing and marketing 3DPF meat alternatives.

### 4.3. 3DPF for Nutrition and Health in Acute or Chronic Conditions

Several studies are being carried out in personalized nutrition to design formulations with specific nutrient quantities to prevent and treat diseases, malnutrition [3], nutritional deficiencies, and allergens while respecting individual food preferences. In addition, individuals with swallowing and chewing difficulties are also a target audience for this new 3DPF technology [2]. This context has been discussed, and, for instance, the project PERFORMANCE (Grant agreement number: 312092) [65] applies 3D printing to produce smooth food for people with chewing or swallowing problems. Moreover, altered food consistency during recovery from surgical interventions, particularly when nutritional intake is fundamental to promoting health and the inclination towards food acceptance is typically diminished, makes 3DPF with adequate consistency a tool for providing essential nutrients and energy.

For instance, cellulose nanocrystal (CNC)-based protein/polysaccharide aqueous foodinks using gelatin B and whey protein isolate for 3DPF, combined with xanthan gum, were developed to create a food for dysphagic individuals [66]. The authors observed that the addition of CNC improved rheological properties, increased fluidity and self-sufficiency, and enhanced thermal stability. The study by Guo et al. [67] developed a formulation using corn flour, wheat flour, and chickpea protein isolate to produce imprinted cereals for dysphagic diets. Different concentrations of chickpea protein isolate were incorporated into the formulations. The authors found that 10% and 20% did not provide a stable impression, and the highest concentrations (30% and 40%) were the most suitable for dysphagia. Similarly, a 3DPF-shaped meal to serve as a nutritious dysphagia-oriented food using different thickeners, such as xanthan gum, guar gum, locust bean gum, Arabic gum, and κ-carrageenan gum, revealed that the highest concentrations of all the thickeners used resulted in a significant increase in yield strength and viscosity of the foodinks [68]. All formulations showed high printing accuracy, excellent self-sustaining capacity, and a smooth surface texture, indicating their potential for use in dysphagic diets.

Despite being a recent technology that is constantly evolving, 3DPF applications still require improvement to achieve the desired product consistency. It is therefore essential to investigate the materials, techniques, and technological procedures to assess their impact on food manufacturing.

On the other hand, because it is a relatively new technique, there are still barriers to overcome in this market. Among them, it is worth mentioning the regulation involved, which has not yet been fully consolidated, generating uncertainties about the permissibility of the various stages, such as the shelf life of the products, the biological alteration of the ingredients at the microscopic level, and the stability of the final product. Another noteworthy critical discussion lies in the food safety of 3DPF [4,66]. These studies underscore the need to ensure the quality and safety of 3DPF before it reaches the market. Implementing good hygiene practices in the handling and manufacturing of 3DPF is crucial, and adjustments such as using foodinks with lower water activity have been suggested. Another strategy involves commercializing innocuous foodinks in recyclable or biodegradable packaging [69].

## 5. Landscapes of Bioactive Compounds in 3DPF

**Figure 3:**
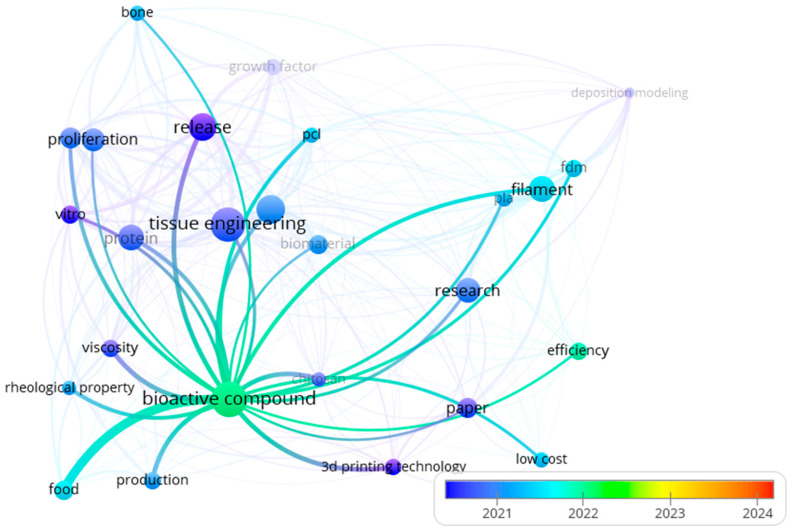
Bioactive compound highlighted in overlay map generated by VOSviewer© 1.6.19 with bibliometric data from Scopus.

Since 2021, publications have begun linking bioactive compounds with 3D printing technology and food, as illustrated in Figure 3, which elucidates recurring terms and their associations found in the titles, abstracts, and keywords of the original papers selected in Section 2. The colors on the map vary according to the publication years of the articles. At the same time, the label sizes and line thickness are directly related to the frequency of term occurrences and the strength of their associations. Thus, “food” has a slightly more recent but stronger association with “bioactive compounds,” as indicated by the line thickness between the highlighted items. Notably, the strength of the association between the terms “food” and “bioactive compounds” may also encompass the fact that foods are sources of bioactive compounds and not necessarily their application in 3DPF systems. Additionally, the occurrence of the terms “bioaccessibility” or “digestibility” was not sufficient for the software to compute and display them as labels in Figure 3.

Therefore, the primary focus on integrating bioactive compounds from fruits, vegetables, roots, algae, insects, and other biodiversity actors into 3DPF further enhances the current technology. The feasibility of this process lies in understanding and adjusting the interactions of bioactive compounds with other foodink ingredients, printing parameters, rheological and textural analysis, and post-treatments (Table 1), as well as antioxidant activity, bioaccessibility parameters, and health effects (Table 2).

Despite variations in printing speed, nozzle diameter, and layer height, successful 3DPF was obtained by controlled deposition of continuous filaments, highlighting extrusion-based systems as the most versatile and accessible technology for structuring bioactive-enriched food formulations. This is a crucial feature directly related to the rheology essays, where viscoelastic and shear-thinning behaviors are desirable and achieved by incorporating biopolymers such as gelatin, starch, gellan gum, pectin, and protein-based ingredients or structured lipid systems (Table 1) [43,44]. In contrast, foodinks incorporating whole matrices, such as fruit pulps, juices, and byproducts, exhibited rheological behavior strongly dependent on solids content, particle size distribution, and matrix complexity [6,70,71,72,73,76]. Higher pulp or flour content generally increased viscosity and elasticity, thereby improving printability [70,71,72], whereas acidic components, such as grape juice, reduced gel firmness by altering protein network interactions [73]. These results demonstrate that whole-matrix formulations introduce additional variables—including pH, particle size, and fiber content—that influence rheological behavior depending on the formulation.

Post-processing, including vacuum drying, oven drying, and air frying, significantly affected the properties of betaine-enriched oat-based 3D-printed snacks. The highest betaine content (1281–1497 mg/g) was observed with the vacuum-drying post-processing method, indicating that temperature is a crucial influencing parameter. Notably, porosity (%) was significantly higher for vacuum-dried samples (40.37 ± 4.44), while firmness (N) differed in air-fried and oven-dried samples (47.57 ± 10.09; 58.32 ± 5.53, respectively) with no statistically significant difference between them [72]. These effects, associated with printing infill ratio and post-processing time, affect the texture. Additional investigation is required to evaluate the stability of bioactive compounds’ antioxidant capacity and their bioaccessibility. The existing literature comprises a few papers assessing the bioaccessibility of bioactive compounds in 3DPF (Table 2), which are discussed as follows.

After careful evaluation of the scientific literature, antioxidant activity was predominantly assessed using standardized in vitro assays, including TPC, DPPH, and FRAP [30,68,70,71,76,79]. In parallel, bioaccessibility assessments were scarce, but when performed, they were mainly conducted using in vitro gastrointestinal digestion models, reflecting the current emphasis on digestive stability and release behavior as key indicators of functional performance in 3DPF systems [16,74,75,76]. Health outcomes were scarce [71] and not standardized.

Starch content affected (*p* < 0.05) contents of condensed tannins, anthocyanins, flavonols, and total flavonoids in up to 15%, except for total phenols and hydroxycinnamic acids. Increasing starch to 15% in 3DPF decreased bioactive compound levels because the strawberry content, the source of these compounds, was lower. However, the findings suggest that incorporating starch at a 15% level is ideal for maintaining the bioactivity of the target compounds [30]. The authors noted that using a printing speed of 14,000 mm.s^−1^, a layer thickness of 3.5 mm, a flow rate of 1.65, and a nozzle height of 4.5 mm for the first layer at room temperature was the best condition for greater antioxidant activity as measured by FRAP. Moreover, they did not conduct a bioaccessibility essay on the final 3DPF product.

Structured matrices, such as oleogels, Pickering emulsions, and hydrogel networks, improved the incorporation of lipophilic bioactive compounds such as carotenoids, primarily through microstructural effects in micelles and reduced oil droplet size, gel network formation, and enhanced lipase accessibility [74,75,76], which led to greater bioaccessibility of the bioactive compounds. The bioaccessibility of commercial fucoxanthin [80] was analyzed in a 3DPF trial [44]. The evaluation was conducted by combining fucoxanthin with isolated whey protein in a high internal phase oleogels-in-water emulsion system. The gel structure obtained with whey protein mixed with glyceryl monostearate (GM) at 6% resulted in increased viscoelasticity of the foodink and reduced droplet size. Consequently, the samples showed enhanced printability and bioaccessibility. After in vitro digestion, the GM-containing samples exhibited a bioaccessibility of 45.48%, while the GM-free sample showed a bioaccessibility of 28.62%. The hydrolysis of GM releases fatty acids, which form micelles to transport fucoxanthin. GM contributed to creating a gel-like network in the foodink, reducing the emulsion droplet size so lipases could act, thereby improving the bioaccessibility of fucoxanthin.

Curcumin, usually extracted from the rhizome of *Curcuma longa* [81] and trans-resveratrol, is found in many biodiversity sources such as seeds, stems, roots, leaves, fruits, and flowers, with a highlight to *Paeonia suffruticosa* flower and roots of *Reynoutria japonica* [82] were co-axial electrospray followed by gelatin hydrogel 3D printing to study the bioaccessibility of these bioactive compounds [70]. Zein combined with polyethylene glycol nanoparticles improved the bioaccessibility of curcumin and resveratrol by 3.6- and 1.7-fold, respectively, and intestinal permeability by 3.5- and 2.2-fold compared with their native forms. The authors achieved exciting results since the 3DPF butterfly shape was stable at room temperature. It is worth mentioning that commercial curcumin and resveratrol were applied; however, there is potential to use them extracted directly from the flora, and the antioxidant activity of the final product was not investigated or assessed by Shang et al. [44].

Li et al. [78] developed a high internal phase emulsion (HIPE) containing 2% wt. pea protein isolate, hyaluronic acid, and tannic acid at a 7:1 ratio, which exhibited the best emulsion stability due to reduced interfacial tension and a more viscoelastic oil-water interfacial profile. Additionally, curcumin-loaded HIPE showed adequate printability, producing a foodink with appropriate rheological parameters, including gel strength and shear-thinning behavior. The bioaccessibility of curcumin was 78.6%; however, the authors suggest further studies to discuss its bioavailability in animal experiments. A bioaccessibility study on 3D-printed grape juices for people with dysphagia found that two- and four-hour dry-heated starch-loaded grape juices increased bioaccessibility (28.7% and 22.9%, respectively) [76]. In contrast, for crude starch, bioaccessibility decreased by 16.3% due to changes in chemical bonding after heat treatment, leading to higher release during in vitro digestion. Thus, gelling and heat treatment affected the bioaccessibility of the anthocyanins.

Encapsulation is an effective strategy to improve the stability and bioavailability of bioactive compounds. Biopolymer-based systems act as encapsulating systems, and their integration with 3D food printing enables control over structure and release features for pre-encapsulated bioactive compounds with adequate protection [78], or for encapsulating compounds during the printing process, for instance, lutein and anthocyanins with enhanced bioaccessibility [83]. The stability of snacks containing 10% starch, 10% ethylcellulose, and 20 mg.g^−1^ lutein, showing that encapsulated lutein exhibited greater retention indexes of approximately 70% and 48% after 21 days of storage at 25 °C and 50 °C, respectively [16]. In contrast, the unencapsulated lutein mixture showed lower retention indexes under the same storage conditions, at 24% and 10%, respectively. Despite its lipophilicity, Scanning Electron Microscope (SEM) images have confirmed that lutein can be successfully encapsulated within the pores of the 3D starch structure through ethanol-mediated deposition.

## 6. Conclusions

Most of the reviewed studies employed commercial extracts containing isolated bioactive compounds, especially carotenoids. The limited use of whole-food matrices, such as fruits or algae, represents a missed opportunity from a circular-economy perspective. When available, bioaccessibility of bioactive compounds is largely formulation-specific and lacks cross-validation across different food matrices. Besides that, there is a lack of integrated assessment frameworks that simultaneously address rheology, microstructure, digestion, and biological responses.

However, more studies are needed to assess the bioaccessibility of the bioactive compounds through the digestive tract. This lack of research draws attention to the need for further investigations into the behavior and efficacy of the bioactive compounds within the context of 3DPF, mainly to understand their ability to withstand the antioxidant properties after processing conditions and their subsequent release and absorption during digestion, depending on the interaction with the other ingredients in the foodink. Addressing this knowledge gap is crucial to ensuring the successful integration of bioactive compounds into 3DPF, as their stability and bioavailability directly affect the potential health benefits of their consumption. Since the composition of foodinks directly affects the mechanical properties of 3DPF, future research should focus on comprehensive studies evaluating foodink formulations, printing parameters, and post-processing treatments to optimize the retention and release of bioactive compounds, ultimately enhancing the nutritional and health-promoting aspects of 3DPF. Additionally, future studies must continue to address sensory attributes and food safety to ensure effective scale-up from lab to market. As technology becomes more sophisticated, a multidisciplinary approach that bridges food science, materials engineering, and nutrition will be essential to fully realize the potential of 3DPF as a tool for personalized, sustainable food production.

## Figures and Tables

**Table 1:** Critical processing of 3DPF containing bioactive compounds, their rheology, and texture behavior.

3DPF Technique	Print Parameters	Foodink Ingredients	Rheological and Texture Behavior	Post Treatment	Reference
Extrusion	500 mm.min^−1^ speed; 0.84 mm nozzle diameter	10% gelatin; 1, 1.5, 2, 2.5% gellan gum	Gellan added to gelatin leads to shear-thinning and self-healing properties	Stored at room temperature	[70]
Extrusion	15 mm.s^−1^ speed; 0.84 nozzle diameter; infill pattern rectilinear 90% density; 40 °C	30.26% *chenpi* decoction; 35% kiwifruit juice; 2.88% pectin	-	Non specified	[71]
Co-axial extrusion	4 mm.s^−1^ speed; 0.4, 0.7, 1 mm layer height; 25 °C core nozzle; 55, 65, 75 °C outer nozzle; 0.01 mPa core nozzle; 0.17 to 0.38 mPa outer nozzle printing pressure; 0.7 mm core nozzle; 1.2 mm outer nozzle diameter	9, 10, 11, 12% *w*/*w* starch; 6, 8, 10% *w*/*v* ethyl cellulose; 20 mg.g^−1^ lutein in ethyl cellulose solution	The best printability is achieved with a 0.7 mm layer height; ethyl cellulose chains organize, leading to low viscosity and shear-thickening behavior at low shear rates and shear-thinning behavior at higher shear rates.	Stored at 4 °C for 48 h, then freeze-dried	[16]
Extrusion	0.024 mL.s^−1^ flow rate; 20 °C; 1.5 mm nozzle diameter	1:0:0, 9:1:0 8.5:0:1.5 porcine plasma protein; pea protein concentrate; soy protein isolate, respectively; 55% glycerol 45% polymer mixture described above	-	Immersion in salt-containing solutions at room temperature for 24 h	[72]
Extrusion	800 mm.s^−1^ speed; 3.5 mm layer thickness; 1.4 flow rate; 6 mm nozzle height first layer; or 14,000 mm. s^−1^ speed; 3.5 mm layer thickness; 1.65 flow rate; 4.5 mm nozzle height first layer; room temperature	10, 15, 20% corn or wheat starch	Starch content in gels is directly proportional to the hardness; the extrusion test describes the texture of the final printed samples more robustly than the penetration test.	Stored at 4 °C	[30]
Extrusion	20 mm.s^−1^ speed; 1.63 mm layer height; 1.63 mm nozzle diameter; 100% rectilinear infill	30, 50, 70% apricot pulp; 5% gelatin bovine	Greater amounts of pulp have led to better printability and higher viscosity; stronger elastic property, and more solid behavior after extrusion	Stored at room temperature	[73]
Fused deposition modeling extrusion	20 mm.s^−1^ speed; 1.2 mm layer height; 25 °C; 1.2 mm nozzle diameter; 100% infill	30, 50, 70% apricot pulp; 3 g fiber orange byproducts; 5% bovine gelatin	Predominantly elastic behavior; enhanced viscoelastic properties upon the introduction of orange by-product and the elevation of apricot pulp percentage; 30% pulp had better extrusion	Stored at room temperature	[74]
Extrusion	1 mm^3^.s^−1^ print speed; 30 °C, 0.84 mm nozzle diameter, 20, 30, or 40% infill	30 g oat flour; 11.5 g defatted hazelnut flour; 6 g rice protein; 6 g oil; 5 g, sugar; 1.7 g betaine; 0.9 g psyllium; 0.5 g cinnamon	The dough presented a viscoelastic behavior with good printability and smooth extrusion	Oven drying at 130 °C for 20, 25, or 30 min; air frying at 180 °C for 5 or 7 min; vacuum drying at 80 °C for 60 or 90 °C	[75]
Extrusion	10 mm.s^−1^ printing speed; 0.84 mm nozzle diameter; 20 °C	5% bovine gelatin and cassava starch; grape juice	Grape juice reduced the pH from 5.6 to 3.8, leading to a decrease in gel firmness	Stored at room temperature	[76]
Extrusion	1 mm^3^.s^−1^ extrusion rate; 1 mm nozzle diameter	10 mL soybean; 0, 2, 4, 6% GM; 5% heat-induced whey protein isolate. The oil phase was 80% of all emulsions	Glyceryl monostearate ≥ 4% resulted in thixotropic recovery of approximately 90%	Stored at room temperature, with freeze–thaw stability tests also performed	[44]
Extrusion	70 mm.s^−1^ printing speed; 150 mm.s^−1^ extrusion rate; 0.86 mm nozzle diameter	Bigels at 30, 50, 70% oleogel concentration; 1% *w*/*w* lecithin or monoglyceride	-	Stored at 4 °C	[77]
Extrusion	2 programs: 8000 mm.min^−1^ or 1400 mm.min^−1^ speed; 3.4- or 3.5-mm printing line thickness; 1.4 or 1.65 flow rate; 4 mm nozzle diameter; 4.5 or 6 mm first layer height	4, 6, 8% *w*/*w* wheat or corn starch; *Arbustus unedo* homogenized fruit	Corn starch resulted in a smaller particle size distribution, and thus, the firmness was higher	Non specified	[6]
Extrusion	1 mm^3^.s^−1^ extrusion rate; 1 mm nozzle diameter; room temperature; 80% infill	2% salmon byproduct protein; 0, 0.25, 0.75, 1% pectin	The gel network between salmon protein and pectin improved the viscoelasticity. 0.75 and 1% pectin formulations were stable in the range of 20–80 °C	Stored at room temperature	[43]
Extrusion	5 mm.s^−1^ printing speed; 0.84 mm nozzle diameter; 90% infill; 25 °C	Pea protein isolate (PPI); hyaluronic acid (HA); tannic acid (TA); curcumin	Better viscosity, viscoelasticity, and thixotropy, when 7:1 (HA:TA)	Stored at room temperature	[78]

**Table 2:** Functional outcomes of 3DPF containing bioactive compounds.

Bioactive Compounds	Source	Antioxidant Activity	Bioaccessibility	Parameters Affecting Bioaccessibility	Health Effects	Reference
Curcumin and resveratrol	Commercial	80% DPPH scavenging activity and 250 μM ascorbic acid FRAP	79% (curcumin); 82% (resveratrol)	Solubility of the ingredients		[70]
Total flavonols; total phenolic content	Jelly made of kiwifruit (*Actinidia chinensis* Planch), commercial pectin, and *chenpi* (aged citrus peel from *Citrus reticulata*)	81.65 ± 2.65 μmol/g TE ABTS and 5.94 ± 0.21 μmol/g TE DPPH radical scavenging capacity, and 15.49 ± 0.21 μmol/g TE FRAP reducing capacity.	-	-	Food intake, liver weight, and adipose tissue weight were significantly reduced in the mouse model	[71]
Lutein	Commercial	-	-	The authors discuss that the amorphous structure of the 3DPF would probably increase the bioaccessibility	-	[16]
Astaxanthin	Commercial	-	-	-	Astaxanthin	[72]
Condensed tannins, hydroxycinnamic acids; anthocyanins; flavanols; total flavonoids	Strawberry juice blend	486.96 ± 0.29 µM DPPH and 1.24 ± 0.01 mM FRAP	-	-	Condensed tannins, hydroxycinnamic acids; anthocyanins; flavanols; total flavonoids	[30]
Total carotenoids, lycopene, and total phenols	Apricot pulp (*Prunus* *Armeniaca* L.)	58.1 ± 1.2 mgGA/100 g of total phenolic compounds (TPC) in the formulation with 70% pulp	-	-	Total carotenoids, lycopene, and total phenols	[73]
Total carotenoids, lycopene, and total phenols	Apricot pulp; orange byproducts	179 ± 14 mgTrolox/100 g of TPC in the formulation with 70% orange byproducts	-	-	-	[74]
Betaine	Commercial	-	-	Although the authors did not measure the bioaccessibility, the temperature affected the total amount of betaine, as the most significant amount was achieved in the vacuum drying post-processing	-	[75]
Anthocyanins	Pasteurized whole grape juice	-	28.7% and 22.9% in heat-treated starch and grape juice; 16.3% without heat treatment	Gelling and heat treatment (130 °C) for 2 h or 4 h to dry the starch before the 3D printing process	All gels reduced NF-κB activation, while TNF-α and CXCL2/MIP-2 secretion decreased in cells treated with native and starch-dried gels for 2 h before obtaining 3D printed gels	[76]
Fucoxanthin	Commercial	-	45.48% in the samples with GM and 28.62% without GM	Encapsulation of fucoxanthin by oleogels-in-water HIPEs. In the microstructure, GM hydrolysis and gel network enhanced micelle formation and reduced lipid droplet size	-	[44]
Quercetin and catechin	Commercial	-	53.37% (catechin); 11.08% (quercetin)	GM and lecithin; oil droplet size and hydrogel-in-oleogel structure enhanced the digestion of lipids	-	[77]
Tannins, hydroxycinnamic acids, flavonols, total flavonoids, and anthocyanins	*Arbutus* *Unedo* L. extract	652.86 ± 10.59 (mg GAE/100 g) TPC; 67.82 ± 0.06 (mg TE/100 g) DPPH; 1.49 ± 0.03 (g TE/100 g) FRAP for the formulation containing 4% starch	-	Lee speed, flow, line thickness, and nozzle diameter did not affect DPPH, but affected FRAP	The effects (protection against DNA damage, cytotoxic effects against the carcinogenic AGS cell line, and HepG2) were evaluated in the extracts and not in the final 3DPF	[6]
Fucoxanthin	Commercial	80.75% DPPH radical scavenging in the sample containing 1% pectin	50.06% in the sample containing 1% pectin	The amount of pectin contributed to a stronger spatial resistance, avoiding the accumulation of droplets and favoring the lipase action; the Pickering emulsion gels slowed the release of fucoxanthin within the intestinal fluid; both mechanisms enhanced bioaccessibility	-	[43]
Curcumin	Commercial	-	78.6% when 7:1 (HA:TA)	Gel network structurization by HA and TA, and a smaller oil droplet size		[78]

## Data Availability

No new data were created or analyzed in this study. Data sharing is not applicable to this article.
